# Effects of a Combined Antimicrobial Peptide and *Candida utilis* Supplementation on Rumen Development and Microecology of Weaned Hu Lambs

**DOI:** 10.3390/microorganisms14091982

**Published:** 2026-09-08

**Authors:** Qing Lin, Jinjuan Zhao, Xiangdong Huo, Kai Lou, Qidong Zhu, Ruoyu Mao, Min Hou

**Affiliations:** 1 Institute of Microbiology, Academy of Agricultural Sciences of Xinjiang Uyghur Autonomous Region, Urumqi 830091, China; qinglinxj@xaas.ac.cn (Q.L.); huoxd@xaas.ac.cn (X.H.); loukai@tsinghua.org.cn (K.L.); qidongzhu@foxmail.com (Q.Z.); 2 Xinjiang Laboratory of Special Environment Microbiology, Urumqi 830091, China; 3 College of Animal Science, Xinjiang Agricultural University, Urumqi 830052, China; 18189678475@163.com; 4 Institute of Feed Research, Chinese Academy of Agricultural Sciences, Beijing 100080, China

**Keywords:** weaned Hu lambs, *Candida utilis*, antimicrobial peptides, rumen development, metabolomics, 16S rRNA sequencing

## Abstract

Rumen development and microecological homeostasis are critical for post-weaning health in lambs; however, effective nutritional strategies combining probiotics and antimicrobial peptides remain underexplored. This study investigated the effects of graded doses of a combined additive (*Candida utilis* + antimicrobial peptide (AMP)) on rumen development and microecology in weaned Hu lambs. Forty healthy pre-weaning male lambs (initial body weight: 15.77 ± 1.25 kg; age: 42.98 ± 1.80 d) were randomly assigned to four groups (*n* = 10): a control group (CK, fed milk/basal diet), and three treatment groups receiving the same milk/basal diet supplemented with graded doses of the combined additive, based on feed intake (LCB: AMP 0.05 g/kg + *C. utilis* 0.1 g/kg, MCB: AMP 0.05 g/kg + *C. utilis* 0.8 g/kg, HCB: AMP 0.05 g/kg + *C. utilis* 1.5 g/kg) over a 40-day period starting from day 6. At the end of the trial, six lambs per group were randomly selected for sampling for analyses of rumen morphology, fermentation parameters, 16S rRNA amplicon sequencing, and untargeted metabolomics. Results showed that, compared with CK, (1) MCB and HCB increased papilla height, and HCB also increased papilla width (*p* < 0.05); papilla width and muscular layer thickness showed significant linear increases with increasing additive dose (*p* < 0.05). (2) All treatments linearly reduced ruminal pH (*p* < 0.05), although all values remained within the normal physiological range; meanwhile, all treatment groups showed elevated levels of microbial protein (MCP), butyrate, valerate, and isovalerate; MCP also increased linearly with dose (*p* < 0.05); LCB decreased the acetate-to-propionate ratio(A:P), and this ratio exhibited a significant quadratic trend across treatment groups (*p* < 0.05). (3) HCB increased the α-diversity indices and reduced Methanobacteriota abundance; LCB elevated Bacillota; and all treatments decreased Spirochaetota, Synergistota, and Cyanobacteriota but increased Actinomycetota, Verrucomicrobiota, and Thermodesulfobacteriota (*p* < 0.05). (4) The number of differential metabolites and KEGG pathway enrichments gradually increased with increasing doses of the combined additive, with HCB exerting the most extensive modulatory effects on signaling and neural pathways. (5) Volatile fatty acids, Bacteroidota, and *Xylanibacter* were positively correlated with rumen development parameters (*p* < 0.05). Collectively, dietary supplementation with the combined additive improved rumen fermentation, microbial community structure, and metabolic profiles, with the high-dose (HCB) regimen showing the most effects on select parameters, whereas the low-dose (LCB) regimen exhibited advantages in specific fermentation traits. These findings provide preliminary evidence for the potential application of this combined additive in post-weaning lamb diets.

## 1. Introduction

With the sustained growth of consumer demand for mutton, China’s meat sheep industry is accelerating its transition toward intensification and large-scale production [1]. Early weaning, as one of the key technologies for improving reproductive efficiency, facilitates the recovery of maternal body condition and the adaptation of young lambs to solid feed, thereby shortening the reproductive cycle of ewes and gaining widespread application in industrial practices [2]. However, early weaning is accompanied by maternal–offspring separation and abrupt changes in feeding patterns, which induce both psychological and nutritional stress in the lambs [3]. This dual stress can compromise intestinal barrier function, antioxidant defense systems, and immune competence, thereby increasing the risk of diarrhea and mortality [4,5], and significantly affecting ruminal morphological development, fermentation parameters, and the structure and function of the ruminal microbiota [6,7]. The rumen serves as a vital digestive and metabolic organ in ruminants, playing a central role in nutrient metabolism, and the pre- and post-weaning periods represent a critical window for ruminal development [6]. Accumulating evidence indicates that scientific management strategies and precision nutritional interventions are effective approaches for alleviating weaning stress, promoting ruminal development, and enhancing lamb growth performance [8,9].

Within the framework of precision nutritional intervention strategies, antimicrobial peptides (AMPs) and *Candida utilis* have attracted increasing attention as highly promising functional feed additives for livestock and poultry production. Accumulating evidence indicates that AMPs can improve the growth performance of various livestock species through multiple mechanisms, including direct bactericidal activity, enhancement of intestinal barrier function, modulation of immune responses, and improvement of antioxidant and anti-stress capacities [10,11,12]. Furthermore, AMPs have been shown to optimize the composition of ruminal bacterial and protozoal communities, elevate digestive enzyme activities, and promote the production of volatile fatty acids (VFAs), thereby systematically regulating the ruminal microecosystem and significantly enhancing the growth performance of young goats [13]. *C*. *utilis* is rich in nutrients and capable of secreting a variety of digestive enzymes, and is therefore commonly employed as a microbial protein source and a starter culture in fermented feed, providing direct or indirect nutritional support to animals [14,15]. In addition, the cell wall of *C. utilis* is abundant in β-glucans and mannan-oligosaccharides, which serve as immunopotentiators that stimulate or enhance host immune function [16]. Regarding the modulation of ruminal fermentation in ruminants, most existing studies have focused on *Saccharomyces cerevisiae* or its yeast culture products [17,18], whereas research on *C*. *utilis* remains comparatively limited. Although the individual application of AMPs or *C. utilis* in livestock production has been documented, systematic evidence regarding their combination in ruminants remains lacking. Notably, our preliminary data indicate that the co-administration of these two additives alleviates diarrhea and promotes body weight gain in weaned lambs. However, the potential effects of the combined additive on ruminal development and the ruminal microecological environment remain unclear.

Therefore, we hypothesized that dietary supplementation with the combined additive (AMP + *C. utilis*) could improve rumen development in weaned Hu lambs by modulating ruminal microbiota and fermentation function. To test this hypothesis, the combined additive was included in the diets of weaned lambs, and the comprehensive effects on ruminal morphology, fermentation parameters, bacterial community structure, and metabolite profiles were systematically evaluated. This study aimed to provide a theoretical foundation for the nutritional management of early weaned lambs and a reference for the application of AMP-probiotic combinations.

## 2. Materials and Methods

### 2.1. Ethical Statement

All animal procedures in this study were approved by the Institutional Animal Care and Ethics Committee of the Xinjiang Academy of Animal Sciences (approval No. 202510030). The experiment was conducted at the mutton sheep breeding facility of Xinjiang Shangpin Meiyang Technology Co., Ltd., Changji, China. All experimental procedures were performed in strict accordance with the Regulations for the Administration of Laboratory Animals of the People’s Republic of China (2017 Edition) [19].

### 2.2. Test Materials

The lyophilized antimicrobial peptide (AMP) powder was developed and supplied by the Institute of Feed Research, Chinese Academy of Agricultural Sciences. The product had a purity of 93.57% and a moisture content of 2.34%. The minimum inhibitory concentration (MIC) of the AMP against the tested strain *Staphylococcus aureus* ATCC 43300 was 2.33 μg/mL. Based on preliminary animal trials, the optimal supplementation level of this product in lamb diets was determined to be 0.05 g/kg feed intake. The lyophilized preparation of *Candida utilis* was developed by the Institute of Microbiology, Xinjiang Academy of Agricultural Sciences, and contained ≥10^10^ CFU/g viable cells. Based on our preliminary trials, when co-administered with the AMP described above, the minimum effective dose of the *C. utilis* preparation for alleviating bacterial diarrhea in weaned lambs was 0.1 g/kg feed intake; within the range of 1.0–2.0 g/kg feed intake, daily weight gain was significantly improved, whereas no further enhancement of weight gain was observed at doses above this range.

### 2.3. Animals and Experimental Design

Forty healthy suckling Hu male lambs (initial body weight: 15.77 ± 1.25 kg; age: 42.98 ± 1.80 d; *p* > 0.05) were randomly allocated to four groups (*n* = 10): a control group (CK) and three treatment groups (LCB, MCB, and HCB). The three treatment groups received the basal diet (Table 1) supplemented with a combined additive (AMP + *C*. *utilis*) at different dosages: LCB (AMP 0.05 g/kg + *C. utilis* 0.1 g/kg, equivalent to 1.0 × 10^9^ CFU/kg), MCB (AMP 0.05 g/kg + *C. utilis* 0.8 g/kg, equivalent to 8.0 × 10^9^ CFU/kg), and HCB (AMP 0.05 g/kg + *C. utilis* 1.5 g/kg, equivalent to 1.5 × 10^10^ CFU/kg), all calculated based on actual feed intake. The control group received the same basal diet without any additives. All lambs were managed identically with respect to milk provision, feeding, and weaning schedules.

The experiment lasted for 40 days and consisted of three phases: Phase 1 (adaptation period, days 1–5): All lambs remained with their dams for natural suckling and had no access to solid feed. Phase 2 (pre-feeding period, days 6–10): All lambs continued natural suckling and began to consume a basal diet. Phase 3 (formal experimental period, days 11–40): All lambs were weaned on day 11 and thereafter had free access to the basal diet for 30 days. From day 6 onward, the basal diet for the treatment groups was supplemented with the combined additive at the designated proportions, whereas the control group received the unsupplemented basal diet throughout the experiment.

During the feeding period, the amount of feed offered daily and the orts collected the following morning were weighed and recorded for each group to calculate the average daily feed intake per lamb per group. All lambs were fed twice daily (at 10:00 and 16:00) and had free access to water. Routine management, immunization, and deworming were performed according to standard farm protocols. At the end of the experiment (day 40), six lambs per group that remained clinically healthy with normal growth performance were randomly selected for slaughter. Prior to slaughter, the lambs were electrically stunned and promptly exsanguinated. Rumen fluid and tissue samples were collected for analyses of ruminal histomorphology, fermentation parameters, bacterial community composition, and metabolomic profiles. The effective sample size for all subsequent analyses was *n* = 6 per group.

### 2.4. Collection of Rumen Fluid and Rumen Tissue

Rumen samples were collected immediately after slaughter. The rumen contents were filtered through four layers of sterile gauze to obtain rumen fluid. The rumen fluid pH was measured using a portable pH meter. For each lamb, rumen fluid was collected and immediately aliquoted into 20 sterile 5 mL tubes, snap-frozen in liquid nitrogen, and stored at −80 °C until analyses of fermentation parameters, bacterial communities, and metabolomic profiles were performed. Rumen tissue samples were collected separately using a sterile scalpel. Each tissue piece was rinsed repeatedly with physiological saline until no visible digesta remained, and then fixed in 4% paraformaldehyde for subsequent histomorphological examination.

### 2.5. Histomorphological Analysis of Rumen Tissue

Fixed rumen tissues were processed by Wuhan Servicebio Technology Co., Ltd., Wuhan, China. for paraffin embedding, sectioning, and hematoxylin and eosin (H&E) staining. The stained sections were scanned using a PANNORAMIC digital slide scanner (3DHISTECH Ltd., Budapest, Hungary) for morphometric analysis. Using CaseViewer (v2.4) software, five random fields of view per sample were selected at 10× magnification to measure the ruminal papilla height, papilla width, and muscular layer thickness, and five random fields of view at 200× magnification were selected to measure the stratum corneum thickness. All measurements were performed using the Image-Pro Plus (v6.0) software. Papilla height, papilla width, and muscular layer thickness were recorded in millimeters (mm), while stratum corneum thickness was recorded in micrometers (μm). The mean value of each parameter was calculated.

### 2.6. Determination of Rumen Fermentation Parameters

Rumen fluid pH was measured immediately after collection using a portable pH meter (Shanghai INESA Scientific Instrument Co., Ltd., Shanghai, China). The ammonia nitrogen (NH_3_-N) concentration was determined using the colorimetric method improved by Feng and Gao [20] at a wavelength of 700 nm. The microbial protein (MCP) content was quantified using the Coomassie brilliant blue method described by Bradford [21]. Volatile fatty acid (VFA) concentrations were analyzed by Shanghai Biotree Biotech Co., Ltd., Shanghai, China. using gas chromatography-mass spectrometry (GC-MS, Agilent 7890B/5977B, Agilent Technologies, Inc., Santa Clara, CA, USA). Rumen fluid samples (200 μL) were placed into 1.5 mL microcentrifuge tubes and acidified with 0.05 mL of 50% H_2_SO_4_. Subsequently, 0.2 mL of extraction solution containing the internal standard (2-methylpentanoic acid, 25 mg/L in methyl tert-butyl ether) was added. The mixture was vortexed for 30 s, shaken for 10 min, and ultrasonicated in an ice water bath for 10 min. After incubation at −40 °C for 60 min, the samples were centrifuged at 10,000 rpm for 15 min at 4 °C. The resulting supernatant was transferred to an autosampler vial for GC-MS analysis.

### 2.7. Total DNA Extraction from Rumen Microorganisms and Amplicon Sequencing

Rumen fermentation fluid samples stored at −80 °C were sent to Beijing Novogene Bioinformatics Technology Co., Ltd., Beijing, China. for microbiome analysis. Total genomic DNA was extracted from the rumen fluid using the CTAB method. The V3–V4 hypervariable regions of the bacterial 16S rRNA gene were amplified using bacterial-specific primers 341F (5′-CCTAYGGGRBGCASCAG-3′) and 806R (5′-GGACTACNNGGGTATCTAAT-3′). The amplified products were purified, pooled in equimolar amounts, the target fragments were recovered, and libraries were constructed, followed by sequencing on the Illumina NovaSeq 6000 platform. Raw sequencing data were assembled using FLASH (v1.2.11) and quality-filtered using fastp (v0.23.1) and Cutadapt, then imported into the QIIME2 (v202202) platform, where denoising and chimera removal were performed using the DADA2 (v3.11) plugin to generate an amplicon sequence variant (ASV) feature table. Species annotation of bacterial 16S rRNA sequences was performed using the Silva database (v138.1). All sample sequence data were rarefied to the minimum sequencing depth, and the rarefied data were used for alpha diversity and bacterial community species abundance analysis. The functional potential prediction of ASVs was performed using PICRUSt2 (v2.3.0) and annotated against the Clusters of Orthologous Genes (COG) database, and the relative abundances of each functional pathway were extracted for subsequent statistical analysis [22,23].

### 2.8. Untargeted Metabolomics Analysis of Rumen Fluid

Rumen fermentation fluid samples stored at −80 °C were sent to Beijing Novogene Bioinformatics Technology Co., Ltd., Beijing, China. for untargeted metabolomic analysis. Samples were extracted with 80% methanol in water, centrifuged at 15,000× *g* and 4 °C for 20 min, and the supernatants were collected for LC-MS analysis. Raw data were converted to mzXML format using ProteoWizard (v3.0.25329) and XCMS (v4.11.1) was used for peak selection, alignment, and retention time alignment. The peak area of each sample was normalized. Peaks with >50% missing values in all samples were removed. The remaining features were matched against the Novogene in-house library for metabolite identification. Differential metabolites were defined as those with VIP > 1, *p* < 0.05, and |log_2_(FC)| ≥ 0.26. Multivariate statistical analyses were performed on the identified metabolites. The KEGG database (https://www.genome.jp/kegg/pathway.html, accessed on 15 July 2026) was used to investigate the functions and metabolic pathways of the metabolites. A metabolic pathway was considered enriched when x/n > y/n and significantly enriched when *p* < 0.05 [24,25,26].

### 2.9. Statistical Analysis

All data were tested for normality and homogeneity of variances. One-way analysis of variance (ANOVA) followed by Duncan’s multiple range test was performed using SPSS software (v23.0) to determine significant differences among groups. Linear and quadratic contrast analyses were conducted to evaluate dose–response effects across the treatment groups (LCB, MCB, and HCB). The significance level was set at *p* < 0.05. Continuous variables are presented as mean ± standard deviation (SD). Microbial data analyses and figure generation were performed and visualized using the data platform of Beijing Novogene Bioinformatics Technology Co., Ltd., Beijing, China. (https://magic-plus.novogene.com/#/, accessed on 15 July 2026).

## 3. Results

### 3.1. Effects of Different Doses of the Combined Additive on Rumen Morphology

**Figure 1:**
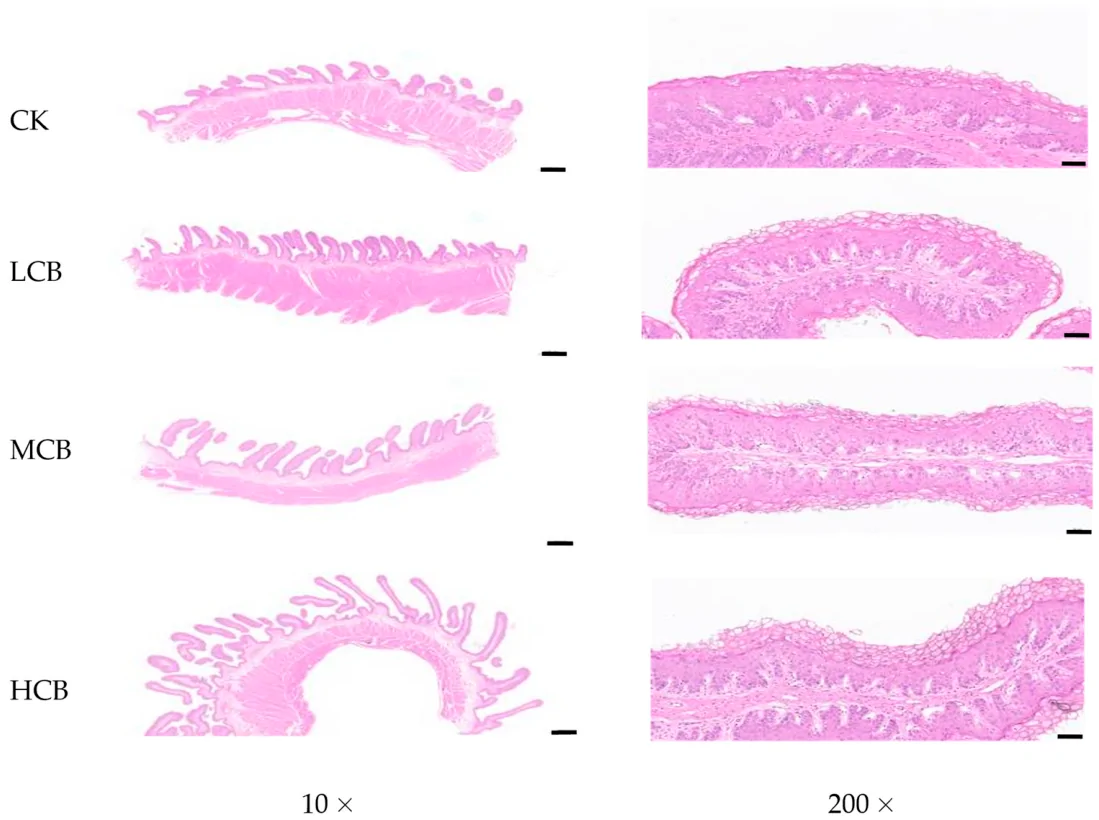
Rumen tissue sections of weaned Hu lambs under different treatments. Note: Scale bars represent 1000 μm at 10× magnification and 50 μm at 200× magnification.

As shown in Figure 1 and Table 2, dietary supplementation with different doses of the combined additive significantly affected rumen development in weaned Hu lambs. Ruminal papilla height tended to increase with additive dosage and was significantly higher in the MCB and HCB groups than in the CK group (*p* < 0.05). Papilla width in the HCB group was significantly greater than that in the other three groups (*p* < 0.05), and a significant linear dose-dependent increase was observed across the treatment groups (*p* = 0.004). No significant differences were observed in muscular layer thickness or stratum corneum thickness across all groups (*p* > 0.05).

### 3.2. Effects of Different Doses of the Combined Additive on Rumen Fermentation Parameters

Rumen fermentation parameters for each treatment group are summarized in Table 3. Ruminal pH was lower in all treatment groups than in the CK group (*p* < 0.01) and decreased linearly with increasing additive dose (*p* = 0.005), although all values remained within the normal physiological range. MCP content increased in all supplemented groups (*p* < 0.05), with a significant linear response to dose (*p* = 0.036), and the highest value was observed in the HCB group. No differences were detected among the groups for NH_3_-N (*p* > 0.05). Butyrate, valerate, and isovalerate concentrations were elevated in all treatment groups compared to those in the CK group (*p* < 0.05). Acetate was reduced only in the LCB group (*p* < 0.05), while the MCB and HCB groups remained comparable to the CK group (*p* > 0.05). Propionate and total VFA levels did not differ significantly among the groups (*p* > 0.05). Among the branched-chain fatty acids, isobutyrate showed a different pattern, with lower values in the LCB and MCB groups than in the CK group (*p* > 0.05). The acetate-to-propionate ratio (A:P) was lower in all treatment groups than in the CK group, and reached significance only in the LCB group (*p* < 0.05). No linear trend was evident across the treatment groups (*p* = 0.350); however, a significant quadratic trend was detected (*p* = 0.035).

### 3.3. Effects of Different Doses of the Combined Additive on Rumen Bacterial Community

#### 3.3.1. Rumen Bacterial α-Diversity Indices

Ruminal bacterial α-diversity indices for each treatment group are presented in Table 4. For community richness, the observed_species, Chao1, and ACE indices in the HCB group were significantly higher than those in the CK group (*p* < 0.05). Although the LCB and MCB groups showed higher values for these three richness indices than the CK group, the differences were not statistically significant (*p* > 0.05). Notably, the Chao1 and ACE indices in the MCB group were slightly lower than those in the LCB group, but no significant difference was detected between the two groups (*p* > 0.05). These results indicate that high-dose combined additive supplementation significantly increased the species richness of the rumen bacterial community in weaned Hu lambs. No significant differences were observed among the groups in the Shannon, Simpson, or PD_whole_tree indices (*p* > 0.05). The goods_coverage values approached 1.00 in all groups, indicating that the sequencing depth adequately covered the bacterial community diversity in each sample and that the data were reliable.

#### 3.3.2. Rumen Microbial Community Composition

**Figure 2:**
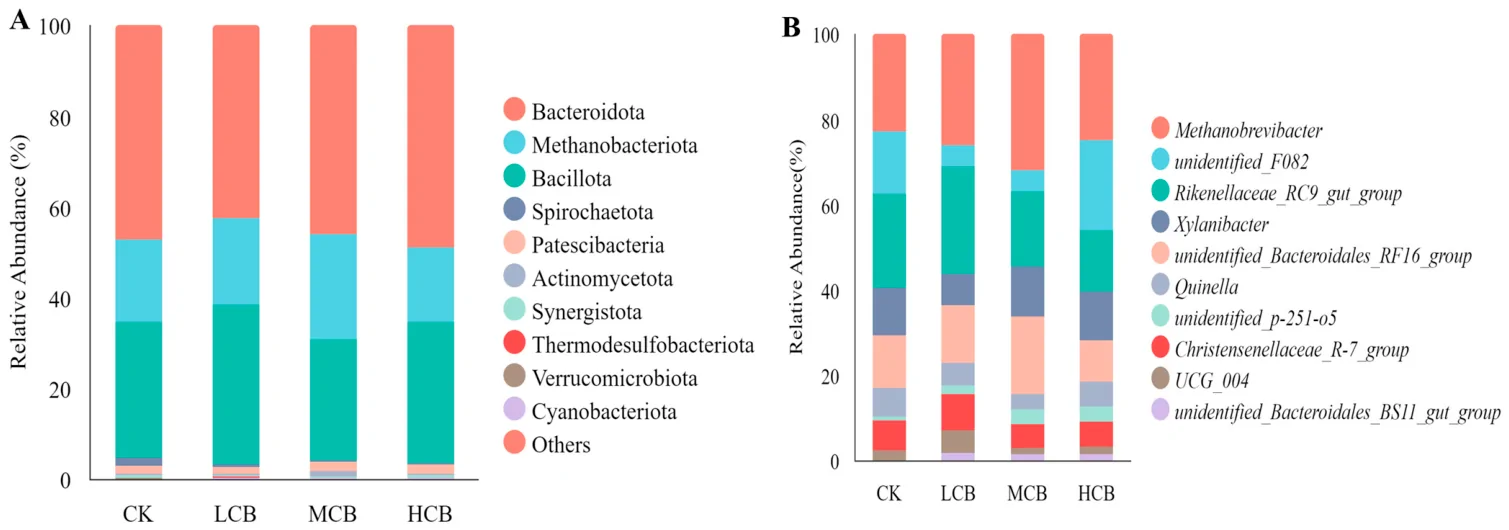
Composition of dominant rumen microbial communities under different treatments. Note: (**A**) Phylum level; (**B**) Genus level.

The top 10 phyla in terms of relative abundance of rumen bacteria are shown in Figure 2A. Bacteroidota, Bacillota, and Methanobacteriota were the dominant phyla across all treatment groups, with their cumulative relative abundance exceeding 85%. Duncan’s multiple range test revealed that Bacteroidota, as the most predominant phylum, reached its highest abundance in the HCB group, which was significantly higher than that in the LCB and MCB groups (*p* < 0.05), but did not differ significantly from the CK group (*p* > 0.05). The abundance of Methanobacteriota was significantly decreased in the HCB group and significantly increased in the MCB group (*p* < 0.05). Bacillota was specifically enriched in the LCB group, and its abundance was significantly higher than that in the CK, MCB, and HCB groups (*p* < 0.05). Among the less abundant phyla, compared with the CK group, all the treatment groups significantly reduced the abundances of Spirochaetota, Synergistota, and Cyanobacteriota, while increasing those of Actinomycetota, Verrucomicrobiota, and Thermodesulfobacteriota (*p* < 0.05). The top 10 genera in terms of relative abundance of rumen bacteria are shown in Figure 2B. *Methanobrevibacter*, unidentified_*f082*, *Rikenellaceae_RC9_gut_group*, and *Xylanibacter* were the predominant microbial genera. The relative abundances at the genus level were generally stable, with most genera exhibiting no significant differences among the groups (*p* > 0.05). Notably, the HCB group significantly increased the abundance of unidentified_*f082* (*p* < 0.05), whereas the MCB group significantly increased the abundance of unidentified_*p-251-o5* (*p* < 0.05). In addition, the LCB group tended to increase the abundances of *Rikenellaceae_RC9_gut_group*, *Christensenellaceae_R-7_group*, and *UCG-004*, whereas the HCB group tended to decrease the abundance of *Methanobrevibacter* (*p* > 0.05).

#### 3.3.3. Predictive Analysis of COG Functional Pathways

**Figure 3:**
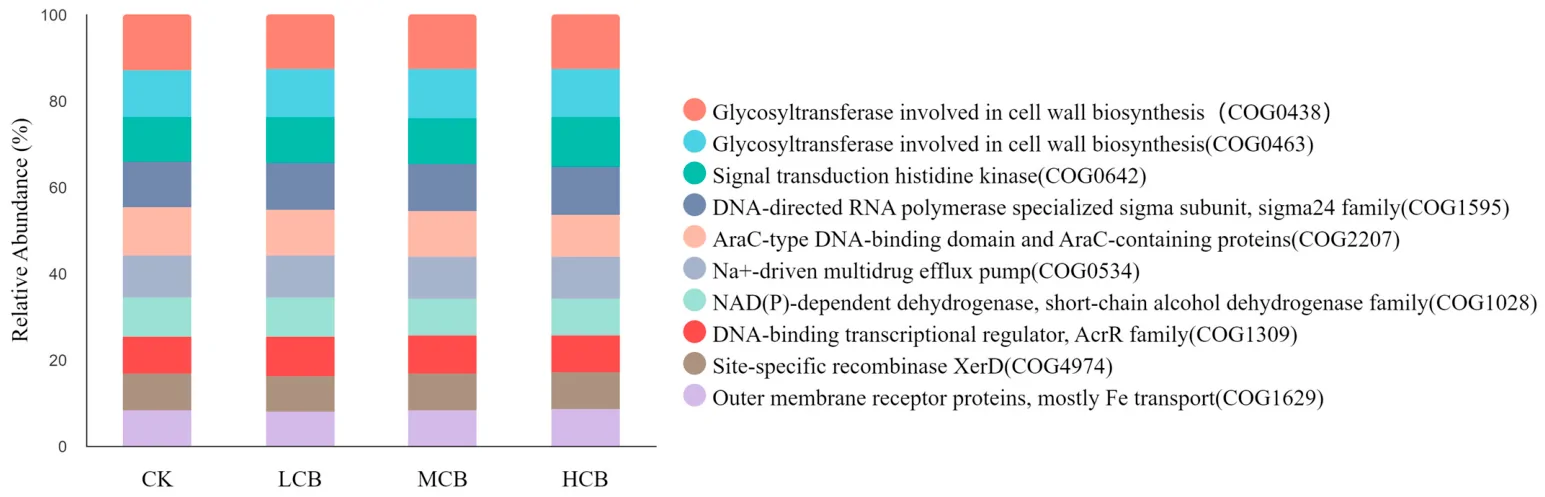
Top 10 COG functional categories of rumen bacteria under different treatment conditions.

Functional gene prediction of the rumen microbial community was performed using PICRUSt2, and the abundance distributions of the top 10 COG functional categories across the treatment groups were comparatively analyzed (Figure 3). The results revealed that the relative abundance profiles of functional categories were highly consistent across all groups, with cell wall/envelope synthesis (COG0438, COG0463), transcription regulation (COG1595, COG2207, COG1309), signal transduction (COG0642), substance transport and drug resistance (COG0534), fundamental redox metabolism (COG1028), DNA recombination (COG4974), and iron uptake (COG1629) as the major functional components. One-way ANOVA followed by Duncan’s multiple range test showed no significant differences among the groups for any of the COG functional categories (*p* > 0.05).

### 3.4. Effects of Different Doses of the Combined Additive on Rumen Fluid Metabolome

#### 3.4.1. Compositional Characteristics and Quantitative Statistics of Differential Metabolites

**Figure 4:**
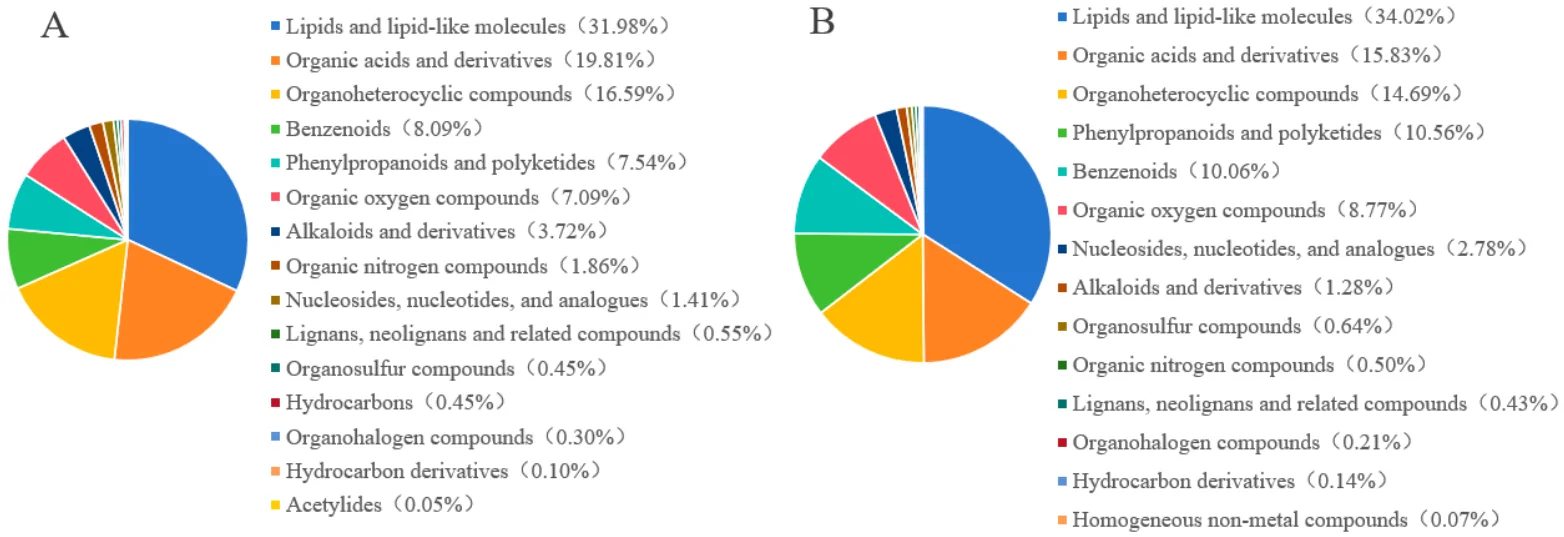
Classification and relative abundance of identified metabolites at Class I level in rumen fluid samples. Note: (**A**) Positive-ion mode; (**B**) Negative-ion mode.

**Figure 5:**
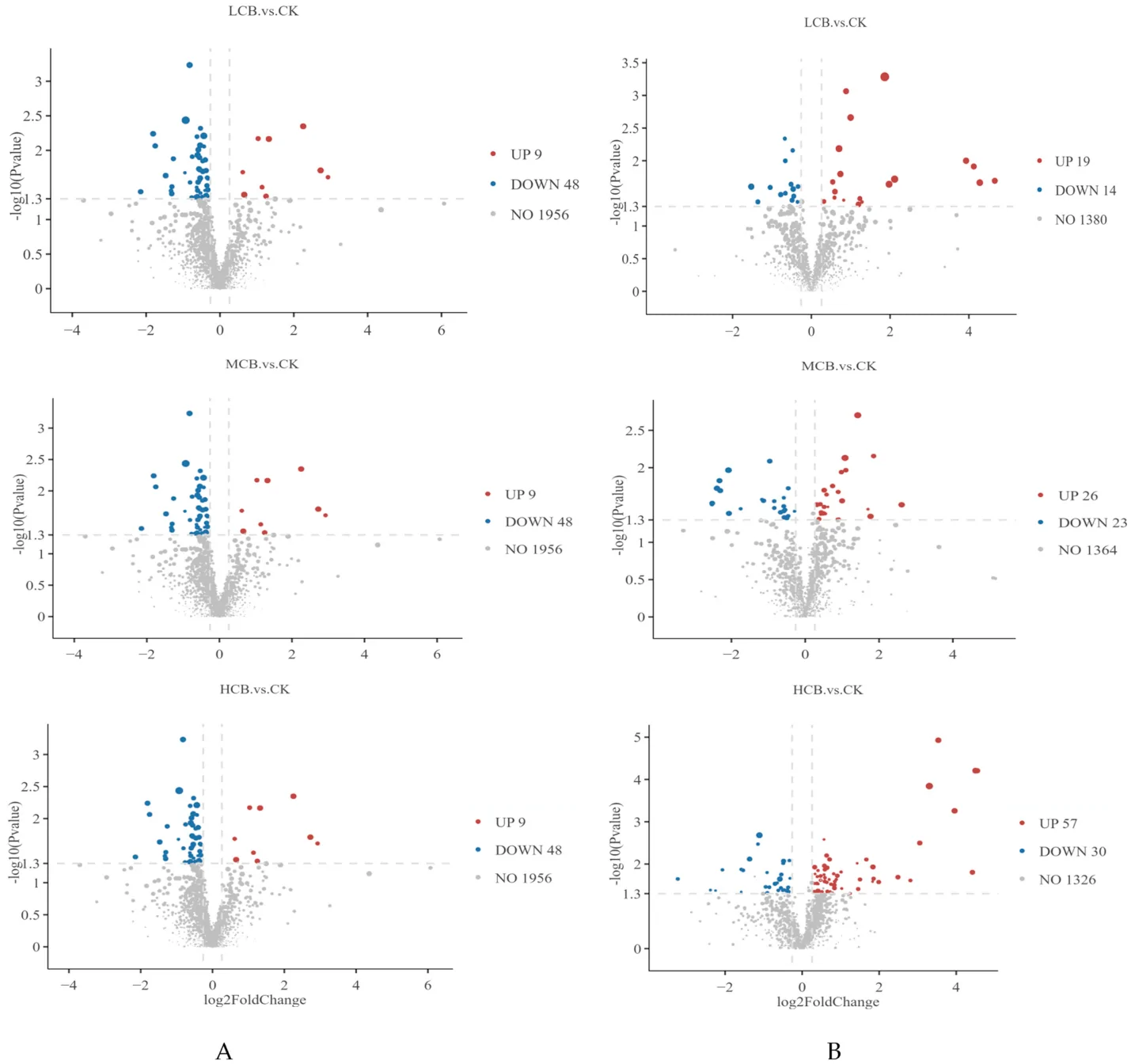
Volcano plots of differential metabolites between each treatment group and the CK group. Note: (**A**) Positive-ion mode; (**B**) Negative-ion mode. The *x*-axis represents the log_2_(FC), and the *y*-axis represents the −log_10_(*p*-value). Red dots represent significantly upregulated metabolites (UP), blue dots represent significantly downregulated metabolites (DOWN), and gray dots represent non-significant metabolites (NO). The filtering criteria were *p*-value < 0.05, |log_2_(FC)| > 0.26, and VIP > 1.0.

Untargeted metabolomics analysis was performed on all rumen fluid samples, and a total of 2013 and 1413 metabolites were identified at Level 1 in positive- and negative-ion modes, respectively. Classification of the detected metabolites revealed that lipids and lipid-like molecules, organic acids and derivatives, and organoheterocyclic compounds were the major classes, each accounting for more than 10% of the total identified metabolites (Figure 4). Further differential metabolite screening was performed for each treatment group using the CK group as the control, followed by volcano plot statistical analysis. The results showed that the number of significantly differential metabolites increased progressively with increasing doses of the combined additive. In the positive-ion mode (Figure 5A), the LCB group exhibited predominantly downregulated metabolites (48 downregulated vs. 9 upregulated); the MCB group showed a reduction in the number of downregulated metabolites (33 downregulated vs. 26 upregulated); and the HCB group displayed a dramatic surge in the total number of differential metabolites to 104 (52 upregulated and 52 downregulated). In the negative-ion mode (Figure 5B), the LCB group showed moderate metabolic activation (19 upregulated vs. 14 downregulated); the MCB group exhibited further activation (26 upregulated vs. 23 downregulated); and the HCB group demonstrated the most pronounced metabolic response (57 upregulated vs. 30 downregulated).

#### 3.4.2. Statistics of Significantly Differential Metabolites

Furthermore, Table 5 summarizes the top 10 significantly differential metabolites in each treatment group relative to the CK group. In the positive-ion mode, the LCB group exhibited upregulation of purine analogues, nitrogenous metabolites, biogenic amines, and plant polyphenols, whereas downregulation was observed for exogenous drug-related compounds, ubiquinones, and various lipids/plant amides. In the MCB group, lignans, amides, alkaloids, and antioxidants were upregulated, whereas amino acid derivatives, steroids, sesquiterpenes, and exogenous drug-related compounds were downregulated. In the HCB group, nitrogenous metabolites, biogenic amines, antioxidants, and bile acids were upregulated, whereas plant alkaloids, branched-chain amino acid peptides, amino acid derivatives, and nucleosides were downregulated. In the negative-ion mode, the LCB group showed upregulation of sulfated phenolic acids, polycyclic ethers, and tricarboxylic acid-related compounds, and downregulation of gallate esters, diterpenoids, glutaric acid derivatives, and Isariotin E. In the MCB group, plant diterpenoids, sulfated phenols, and sesquiterpenes were upregulated, whereas various nucleoside monophosphates and phosphorylated organic acids were downregulated. In the HCB group, sulfated phenolic acids and polycyclic ethers were broadly upregulated, whereas nucleosides and their derivatives, isoprenoids, and phospholipids were downregulated.

#### 3.4.3. KEGG Pathway Enrichment Analysis of Differential Metabolites

**Figure 6:**
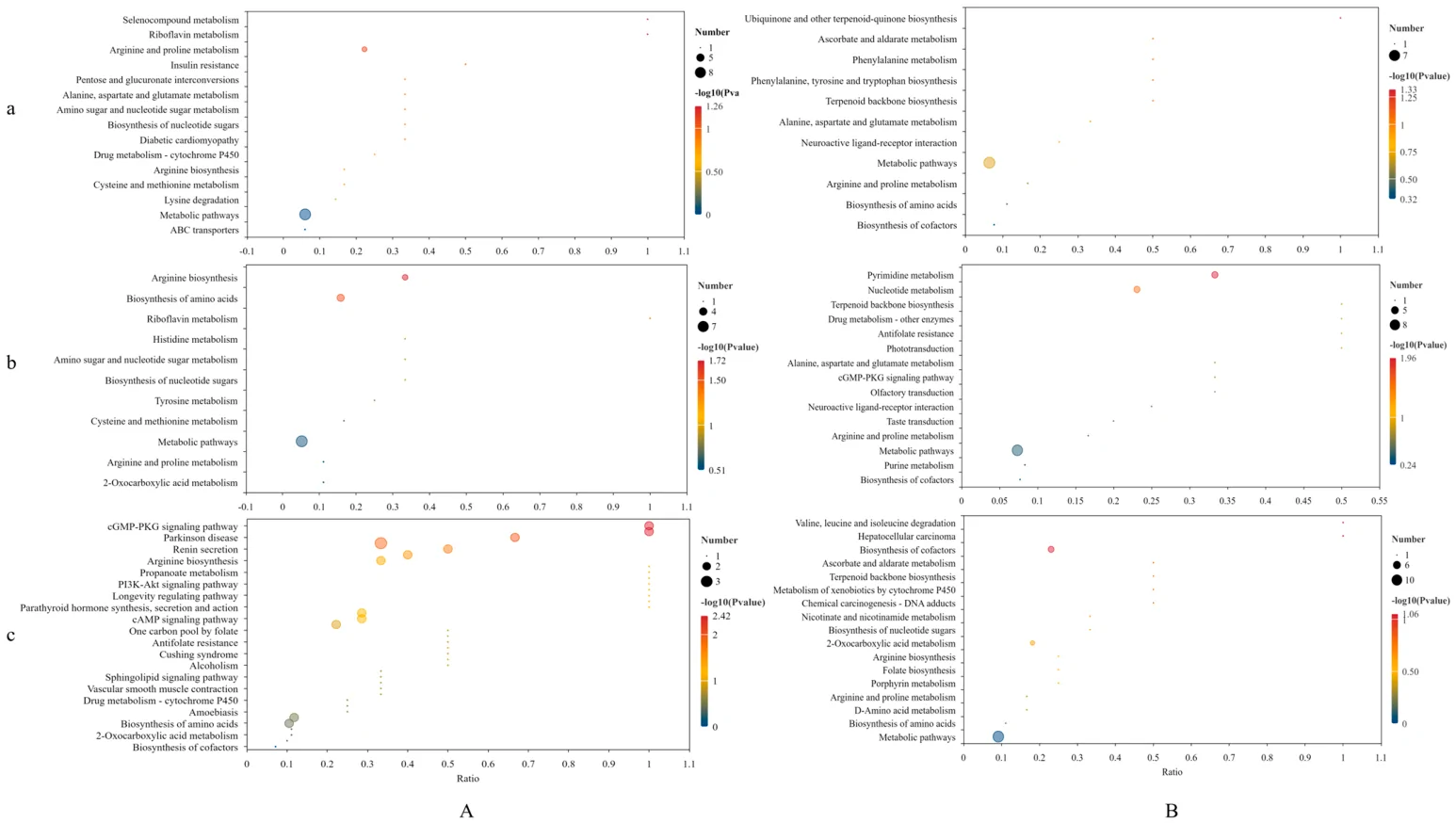
KEGG pathway enrichment analysis of differential metabolites in each treatment group. Note: (**A**) Positive-ion mode; (**B**) Negative-ion mode. The panels correspond to the comparisons: (**a**) LCB vs. CK; (**b**) MCB vs. CK; (**c**) HCB vs. CK. The *y*-axis lists the significantly enriched KEGG pathways. The *x*-axis (Ratio) represents the ratio of the number of differential metabolites to the total identified metabolites within a specific pathway (x/y), where a larger value indicates a higher degree of enrichment. The bubble size represents the number of differential metabolites in the pathway. The bubble color represents the *p*-value calculated by the hypergeometric test (displayed as −log_10_(*p*-value)), with the gradient from blue to red indicating a decreasing *p*-value, reflecting increasing statistical reliability and significance.

Figure 6 shows the KEGG pathway enrichment results for each treatment group relative to the CK group in the positive and negative ion modes. In the positive-ion mode, the LCB group was mainly enriched in selenocompound metabolism and riboflavin metabolism pathways (*p* > 0.05). The MCB group showed significant enrichment (*p* < 0.05) in arginine biosynthesis, amino acid biosynthesis, and riboflavin metabolism. The HCB group showed the broadest pathway coverage, with significant enrichment in the cGMP-PKG signaling pathway and morphine addiction (*p* < 0.01), as well as high enrichment ratios (ratio > 0.4) in Parkinson disease, renin secretion, and regulation of lipolysis in adipocytes. In the negative-ion mode, the LCB group showed significant enrichment in ubiquinone and other terpenoid-quinone biosynthesis, ascorbate and aldarate metabolism, and phenylalanine metabolism pathways (*p* < 0.05). The MCB group showed a different enrichment pattern in the pyrimidine and nucleotide metabolism pathways (*p* < 0.05). The HCB group was mainly enriched in valine, leucine and isoleucine degradation, hepatocellular carcinoma, and biosynthesis of cofactors pathways (*p* > 0.05).

### 3.5. Correlations Among Rumen Morphology, Microbial Community, and Fermentation Parameters

**Figure 7:**
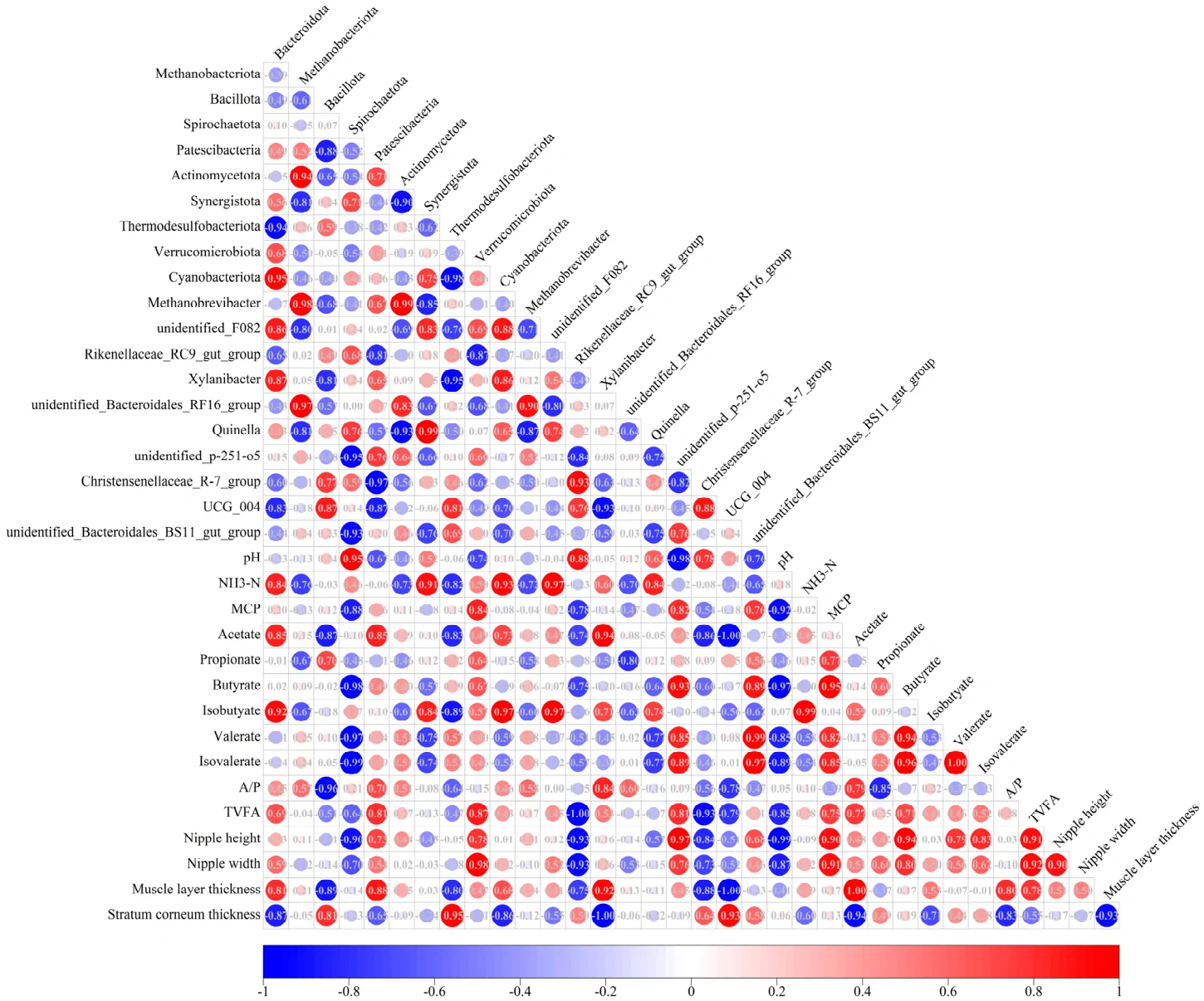
Correlations among ruminal histomorphological parameters, fermentation parameters, and dominant microbial taxa. Note: The numbers within the circles represent the correlation coefficients (*r* values). According to the color scale, red indicates a positive correlation, while blue indicates a negative correlation; darker colors (closer to 1 or −1) indicate stronger correlations. The size of the circles is proportional to the absolute value of the correlation coefficient.

Correlation analyses were performed for histomorphological parameters, dominant microbial taxa (phylum and genus levels), and major fermentation products (Figure 7). Metabolomic data were not included because of inconsistent metabolite profiles across treatment groups. TVFA and butyrate were positively correlated with papilla height and muscular layer thickness (*p* < 0.05 or *p* < 0.01), whereas pH was negatively correlated with these two parameters (*p* < 0.01). Additionally, the three branched-chain VFAs (isobutyrate, valerate, and isovalerate) each showed strong positive correlations with stratum corneum thickness (*p* < 0.01). At the phylum level, Bacteroidota was positively correlated with acetate, isobutyrate, and muscular layer thickness (*p* < 0.05), but negatively correlated with stratum corneum thickness (*p* < 0.05). Methanobacteriota correlated positively with NH_3_-N and several unclassified genera (*p* < 0.05). Cyanobacteriota showed a negative correlation with pH (*p* < 0.05). At the genus level, *Xylanibacter* was positively correlated with acetate and muscular layer thickness (*p* < 0.05), and negatively correlated with stratum corneum thickness (*p* < 0.01). In contrast, *UCG_004* showed a strong negative correlation with both TVFA and muscular layer thickness (*p* < 0.01). *Christensenellaceae_R-7_group* showed significant negative correlations with acetate, TVFA, and muscular layer thickness (*p* < 0.05).

## 4. Discussion

### 4.1. Effects of Different Doses of the Combined Additive on Rumen Histomorphology and Fermentation Parameters in Weaned Lambs

The ruminal histomorphological parameters in this study indicated that at 30 d post-weaning, the rumen of Hu lambs was well developed, and all doses of the combined additive promoted increases in ruminal papilla height and width, with the HCB group showing the most pronounced effects. The weaning period is recognized as a critical window for rumen development, and improper development during this phase can directly impair key physiological functions, including immunity, absorption, and metabolism [27]. Previous studies have established that physical stimulation from solid feed promotes rumen development and functional maturation in lambs [6]. Appropriate feed additives can also facilitate rumen development; for instance, dietary supplementation with antimicrobial peptides significantly increased ruminal papilla diameter and micropapillary density in castrated bulls [28]. The cell wall of *C. utilis* is abundant in mannan-oligosaccharides and β-glucans, which exert immunomodulatory effects by enhancing intestinal barrier function, adsorbing pathogenic bacteria, and stimulating cytokine release [29]. In goats, dietary inclusion of a PEY additive containing yeast cell wall components has been reported to promote rumen development, as reflected by greater rumen weight, larger papillary surface area, and higher expression of genes related to VFA absorption [30]. Notably, in our study, papilla width and muscular layer thickness showed significant linear increases with increasing additive dose, indicating a dose-dependent effect of the combined additive on rumen development. Ruminal pH also decreased linearly with increasing dose, consistent with the trend of increased VFA production, further supporting these dose-dependent effects.

In our study, all three additive groups showed lower ruminal pH than the CK group, although pH remained in the normal physiological range. Concentrations of MCP, butyrate, valerate, and isovalerate increased across treatments, with MCP showing a significant linear increase, and the LCB group showing the lowest A:P ratio (2.70). These findings suggest that different doses of the combined additive all exerted certain positive effects on rumen fermentation. Correlation analysis revealed positive correlations between VFAs and papillary development, consistent with the roles of butyrate in stimulating epithelial proliferation [31] and isovalerate in promoting fiber-digesting bacteria [32]. The increase in MCP across treatment groups may be attributed to the enhanced VFA supply, as VFAs serve as carbon skeletons for microbial protein synthesis, thereby improving nitrogen utilization and providing energy and protein to the host [33]. The A:P ratio below 3.0 in both LCB and HCB suggests a shift to propionate-type fermentation, which improves energy efficiency [34]. The significant quadratic trend for A:P further suggests that among the doses tested, the lowest dose (LCB) may be most favorable for promoting propionate-type fermentation.

### 4.2. Effects of Different Doses of the Combined Additive on Rumen Microbial Community in Weaned Lambs

Bacteria are the most abundant, diverse, and metabolically active component of the rumen microbiota [35]. In our study, the HCB group showed higher bacterial diversity indices and lower Methanobacteriota abundance than the control, whereas the LCB group enriched Bacillota. The LCB and MCB groups had no significant effect on rumen bacterial α-diversity, whereas the HCB group significantly enhanced observed_species, Chao1, and ACE indices. The lack of significant changes in α-diversity in the LCB and MCB groups is consistent with previous findings that long-term (76-day) supplementation with 1.0 × 10^9^ CFU/day of live yeast did not alter α-diversity in artificially reared lambs [36]. In contrast, the yeast–microalgae mixture significantly increased the Chao1, Shannon, and InvSimpson indices in camels but significantly reduced the Chao1 index in sheep without affecting the Shannon and InvSimpson indices [37]. Collectively, these findings suggest that the influence of the combined additive on rumen bacterial α-diversity in the present study appears to be associated with dosage level, host species, and the composition of the additive.

In the present study, the dominant phyla were Bacteroidota, Bacillota, and Methanobacteriota. Bacteroidota and Bacillota were also dominant phyla in previous studies on Hu lambs [38]. These two phyla are reported to degrade dietary carbohydrates to produce propionate and to decompose cellulose, respectively, thereby providing energy and facilitating nutrient absorption for the host [39]. This suggests that the rumen of weaned Hu lambs has established a sufficient microbial community capable of fermenting various types of dietary carbohydrates. However, the predominance of Methanobacteriota may be related to the dietary composition in the present study. It is well established that Methanobacteriota can utilize H_2_ and CO_2_ generated from fiber degradation to produce methane (CH_4_), thereby preventing H_2_ accumulation, which would otherwise inhibit normal rumen fermentation [40]. The diet in the present study contained 44% silage and 16% alfalfa, providing a relatively high-fiber content, which theoretically favors the growth of Methanobacteriota. Notably, the abundance of Methanobacteriota and *Methanobrevibacter* was significantly reduced in the HCB group despite the high-fiber diet, suggesting that the combined additive may have counteracted the fiber-induced enrichment of methanogens. This inhibitory effect is directionally consistent with a previous report that increasing the supplementation level of *C. utilis* in lactating Holstein cows significantly decreased methane production [41], although that study used *C. utilis* alone rather than the combined additive. Furthermore, all treatment groups in this study significantly reduced the relative abundances of Spirochaetota, Synergistota, and Cyanobacteriota, while significantly increasing those of Actinomycetota, Verrucomicrobiota, and Thermodesulfobacteriota. Previous studies have indicated that certain members of Spirochaetota are potential pathogens in the animal gut [42], while a reduction in Synergistota abundance may be linked to decreased ruminal methane production [43]. Actinomycetota include several beneficial bacteria, such as Bifidobacterium, which contribute to gut health through immune modulation, inhibition of pathogen colonization, regulation of microbial interactions, and production of beneficial metabolites [44]. Thermodesulfobacteriota have the potential to reduce hydrogen sulfide concentrations in the rumen, thereby mitigating their toxic effects on ruminal epithelial cells [45]. Moreover, *Akkermansia muciniphila*, a member of Verrucomicrobiota, has been reported to modulate host immunity, maintain intestinal barrier integrity, and participate in the regulation of neuroimmune homeostasis [46]. Collectively, these findings suggest that the combined additive may exert its beneficial effects by inhibiting potential pathogens and methanogen-related taxa, promoting the enrichment of beneficial bacteria, and modulating ruminal sulfide metabolism. Although the combined additive altered microbial diversity and community composition, the COG functional predictions indicated that the overall gene profiles remained stable across all treatments. This stability probably reflects the functional redundancy of the rumen microbiota, which allows digestive functions to be maintained despite dietary or management changes [47].

### 4.3. Effects of Different Doses of the Combined Additive on the Rumen Metabolic Profile in Weaned Lambs

In this study, untargeted metabolomics revealed that the combined additive significantly altered the rumen fluid metabolic profile at all three dose levels, with the number and chemical classes of differential metabolites changing progressively with increasing dose. In the positive-ion mode, creatinine was significantly upregulated in both the LCB and HCB groups, with a greater magnitude of increase in the HCB group. Creatinine is a non-enzymatic dehydration product of creatine and phosphocreatine, and its production rate is closely associated with phosphocreatine turnover and energy metabolism. When cellular energy demand increases, phosphocreatine is rapidly degraded to maintain ATP levels, resulting in enhanced creatinine production [48], suggesting enhanced energy metabolism or accelerated cellular turnover in ruminal epithelial cells in the LCB and HCB groups. In the negative-ion mode, p-coumaric acid sulfate, dihydroferulic acid-4-O-sulfate, and 3-(3-hydroxyphenyl) propionic acid sulfate were upregulated by several- to tens-of-fold across the treatment groups. These compounds are sulfated conjugates of phenolic acids, likely derived from the degradation and sulfation of phenolic acids in plant cell walls by rumen microorganisms. Rumen bacteria can adapt to and metabolize modified phenolic acid compounds, converting them into less toxic derivatives [49]. The pronounced upregulation of these sulfated phenolic acid conjugates is not merely a passive accumulation of terminal metabolites. Rather, it may reflect an active microbial detoxification process: phenolic acids at relatively high concentrations can inhibit certain rumen microorganisms, and their sulfation is considered an important modification mechanism that reduces bioactivity and promotes excretion [50]. Multiple nucleotides (GMP, 2′-deoxyadenosine-5′-monophosphate, and cytidine-3′-monophosphate) in the MCB group and the nucleoside (guanosine) in the HCB group were downregulated. Nucleotides are essential substrates for microbial growth, proliferation, and energy metabolism [51]. The downregulation of these nucleotide-related metabolites, accompanied by elevated MCP levels, suggests that nucleotide synthesis pathways may have been suppressed, while protein synthesis was relatively enhanced. Given that the combined additive contains live yeasts, which can compete nutritionally with the autochthonous rumen microbiota [52], we speculate that medium and high doses of the combined additive may have altered ruminal carbon flow, directing more carbon skeletons toward basal metabolism and protein synthesis rather than de novo nucleotide biosynthesis. Admittedly, this speculation warrants further validation via metabolic flux analysis.

KEGG enrichment analysis in positive-ion mode showed distinct pathway profiles across the three treatment levels. The low dose was mainly linked to antioxidant and coenzyme pathways; the medium dose shifted toward nitrogen metabolism; and the high dose involved broader systemic regulation. This suggests a gradual shift in the primary target: from ruminal microbial metabolism at lower doses to host-level physiological effects at the higher dose. The enrichment of selenocompound and riboflavin metabolism pathways in the LCB group may be attributed to the antioxidant potential of B vitamins and β-glucan derived from *C. utilis* [53]. The significant enrichment of amino acid biosynthesis pathways in the MCB group is consistent with a previous report that yeast products can enhance the assimilation and utilization of ammonia nitrogen by rumen microorganisms, thereby reducing nitrogen loss [54]. This indicates that microbial de novo amino acid synthesis pathways were activated in the MCB group, potentially promoting the efficient conversion of nitrogen into microbial protein. The significant enrichment of the cGMP-PKG signaling pathway and endogenous opioid pathways, together with high ratios for Parkinson disease, renin secretion, and regulation of lipolysis pathways in the HCB group, does not necessarily indicate pathological processes, as the cGMP-PKG signaling pathway exerts anti-inflammatory and barrier-protective effects [55]. Similar pathway changes have been reported in studies of probiotic intervention in inflammatory bowel disease, where the cGMP-PKG pathway played a central role through improved intestinal perfusion and mucosal repair [56]. Given the functions of these pathways, the HCB regimen may have enhanced ruminal papilla growth by increasing local blood flow, modulating neuroendocrine balance, and improving energy metabolism.

### 4.4. Limitations of the Study

One limitation of this study is the absence of groups receiving AMP or *C. utilis* alone. The observed effects therefore reflect the combined additive as a whole, rather than the specific contribution of either component or their interaction. Although the graded responses across the LCB, MCB, and HCB groups suggest a dose-dependent effect, they do not allow us to separate the relative contributions of AMP and *C. utilis*. Future studies with single-additive control groups would help clarify the underlying mechanisms. In addition, we focused on ruminal histomorphology, fermentation parameters, and microbial community structure in weaned lambs. Growth performance, nutrient digestibility, and slaughter traits were not examined, which limits our ability to evaluate the practical value of this additive in production. Future work integrating performance data, nutrient utilization, and blood metabolic parameters would provide a more complete assessment of its application potential and mechanisms of action.

## 5. Conclusions

Dietary supplementation with graded doses of the combined additive improved ruminal fermentation and epithelial development in weaned Hu lambs, and induced dose-dependent changes in ruminal microbial community structure and metabolic activity. Among the doses tested, the high-dose combination (AMP 0.05 g/kg + *C. utilis* 1.5 g/kg, equivalent to 1.5 × 10^10^ CFU/kg) showed the most pronounced promoting effects on microbial diversity and rumen development, as well as the greatest reduction in methanogen abundance. However, the low-dose combination (AMP 0.05 g/kg + *C. utilis* 0.1 g/kg, equivalent to 1.0 × 10^9^ CFU/kg) performed best in reducing the A:P and enriching Bacillota, suggesting that the optimal dose may vary depending on the target parameter. These findings provide preliminary experimental evidence for the potential application of this combined additive in post-weaning lamb diets, although its long-term effects and performance under larger-scale production conditions remain to be confirmed.

## Figures and Tables

**Table 1:** Composition and nutrient levels of the basal diet.

Item	Content
Ingredients (as-fed basis) %	
Lamb concentrate supplement	36.92
Silage	44.05
Alfalfa	16.17
CaHPO_4_ Premix ^(1)^	1.08 1.00
Baking soda	0.52
NaCl	0.26
Total	100.00
Nutrient composition ^(2)^ (dry matter basis) %	
Crude protein (CP)	19.69
Crude fiber (CF)	12.61
Ether extract (EE)	3.51
Neutral detergent fiber (NDF)	30.21
Acid detergent fiber (ADF)	13.84
Acid detergent lignin (ADL)	0.53
Ash	8.37
Calcium (Ca)	1.34
Phosphorus (P)	0.76
Metabolizable energy (ME, MJ/kg)	10.13

Note: ^(1)^: The premix provided the following nutrients per kilogram: vitamin A 8500 IU, vitamin D 2080 IU, vitamin E 25.00 mg, niacin 50.00 mg, biotin 0.15 mg, Cu 12.00 mg, Fe 40.00 mg, Zn 55.00 mg, Mn 65.00 mg, I 0.85 mg, Se 0.05 mg. ^(2)^: The basal diet contained 62.70% dry matter (DM) on an as-fed basis.ME was calculated based on the Nutrient Requirements of Meat Sheep (NY/T 816—2021); all other values were measured.

**Table 2:** Rumen histomorphological parameters of Hu lambs under different treatments.

Items	CK	LCB	MCB	HCB	*p*-Value		
					Treatment	Linear	Quadratic
Nipple height (mm)	1.82 ± 0.16 ^b^	1.95 ± 0.21 ^ab^	2.16 ± 0.41 ^a^	2.23 ± 0.17 ^a^	0.047	0.111	0.650
Nipple width (mm)	0.63 ± 0.10 ^b^	0.66 ± 0.10 ^b^	0.73 ± 0.09 ^b^	0.87 ± 0.13 ^a^	0.004	0.004	0.498
Muscle layer thickness (mm)	1.88 ± 0.16	1.75 ± 0.10	1.94 ± 0.15	1.92 ± 0.12	0.110	0.041	0.124
Stratum corneum thickness (μm)	51.47 ± 13.20	63.86 ± 17.96	52.86 ± 18.72	53.39 ± 17.06	0.572	0.328	0.530

Note: In the same row, values with different lowercase superscripts differ significantly (*p* < 0.05), whereas those with the same or no superscript indicate no significant difference (*p* > 0.05). The same applies below.

**Table 3:** Rumen fermentation parameters of weaned Hu lambs under different treatments.

Items	CK	LCB	MCB	HCB	*p*-Value		
					Treatment	Linear	Quadratic
pH	6.21 ± 0.02 ^a^	6.14 ± 0.02 ^b^	6.07 ± 0.05 ^c^	6.04 ± 0.07 ^c^	<0.010	0.005	0.384
NH_3_-N (mg/dL)	22.84 ± 2.29	22.25 ± 2.10	22.25 ± 1.12	22.84 ± 1.21	0.877	0.517	0.707
MCP (mg/mL)	0.75 ± 0.16 ^c^	0.97 ± 0.21 ^b^	0.98 ± 0.16 ^b^	1.19 ± 0.12 ^a^	0.002	0.036	0.235
TVFA/(mmol/L)	92.10 ± 2.79	90.52 ± 5.85	97.87 ± 4.67	101.05 ± 6.15	0.104	0.061	0.617
Acetate/(mmol/L)	57.96 ± 2.26 ^a^	51.25 ± 1.56 ^b^	59.91 ± 2.20 ^a^	59.39 ± 3.30 ^a^	0.008	0.007	0.039
Propionate/(mmol/L)	17.27 ± 1.25	19.29 ± 3.25	17.22 ± 2.43	19.96 ± 2.32	0.438	0.772	0.254
Butyrate/(mmol/L)	12.14 ± 0.92 ^b^	14.59 ± 1.30 ^a^	15.23 ± 1.82 ^a^	15.94 ± 0.65 ^a^	0.028	0.264	0.970
Isobutyate/(mmol/L)	1.95 ± 0.13	1.66 ± 0.36	1.72 ± 0.08	1.97 ± 0.08	0.204	0.127	0.558
Valerate/(mmol/L)	1.53 ± 0.19 ^b^	1.94 ± 0.11 ^a^	1.96 ± 0.12 ^a^	1.95 ± 0.10 ^a^	0.011	0.960	0.883
Isovalerate/(mmol/L)	1.25 ± 0.16 ^b^	1.79 ± 0.11 ^a^	1.84 ± 0.18 ^a^	1.84 ± 0.19 ^a^	0.006	0.693	0.823
A:P	3.36 ± 0.13 ^a^	2.70 ± 0.37 ^b^	3.52 ± 0.44 ^a^	2.99 ± 0.20 ^ab^	0.046	0.350	0.035

Note: MCP, microbial protein; TVFA, total volatile fatty acids; A:P, acetate-to-propionate ratio. In the same row, values with different lowercase superscripts differ significantly (*p* < 0.05), whereas those with the same or no superscript indicate no significant difference (*p* > 0.05).

**Table 4:** α-Diversity indices of rumen bacterial community in weaned Hu lambs under different treatments.

Items	CK	LCB	MCB	HCB	*p*-Value		
					Treatment	Linear	Quadratic
Observed_species	822.83 ± 95.54 ^b^	901.33 ± 70.65 ^ab^	875.17 ± 126.19 ^ab^	992 ± 87.24 ^a^	0.046	0.128	0.163
Shannon	5.78 ± 0.89	5.85 ± 0.31	5.58 ± 0.88	6.00 ± 0.70	0.799	0.703	0.324
Simpson	0.93 ± 0.05	0.94 ± 0.01	0.91 ± 0.08	0.91 ± 0.06	0.767	0.431	0.580
Chao1	880.05 ± 86.39 ^b^	964.84 ± 75.91 ^ab^	934.77 ± 129.96 ^b^	1083.21 ± 134.51 ^a^	0.030	0.099	0.146
ACE	884.66 ± 90.41 ^b^	966.26 ± 72.3 ^ab^	936.82 ± 127.1 ^b^	1071.53 ± 114.31 ^a^	0.035	0.109	0.146
Goods_Coverage	0.999 ± 0.000	0.999 ± 0.001	0.999 ± 0.000	0.998 ± 0.001	0.352	0.645	0.195
PD_whole_tree	63.72 ± 11.76	61.08 ± 2.58	61.62 ± 5.9	68.65 ± 8.05	0.347	0.044	0.293

Note: In the same row, values with different lowercase superscripts differ significantly (*p* < 0.05), whereas those with the same or no superscript indicate no significant difference (*p* > 0.05).

**Table 5:** Top 10 differential metabolites in each treatment group relative to the CK group.

Contrast	Name	Ion Mode	log2FC	*p*-Value	VIP	Up/Down
LCB. vs. CK	(+)-EHNA	P	2.93	0.02	1.81	up
	CREATININE	P	2.73	0.02	2.43	up
	n-Pentadecylamine	P	2.25	0.00	2.54	up
	Hispidol	P	1.32	0.01	2.48	up
	Tetradeca-2,4,6-trienoylcarnitine	P	1.25	0.05	2.16	up
	Moveltipril	P	−1.33	0.04	1.82	down
	ubiquinone 25	P	−1.47	0.02	2.42	down
	Ridleyamide	P	−1.75	0.01	2.19	down
	N-Linoleoyl Methionine	P	−1.81	0.01	2.31	down
	Meropenem	P	−2.15	0.04	2.16	down
MCB. vs. CK	Secoisolancifolide	P	6.82	0.02	2.01	up
	N-[2,6-Di(propan-2-yl)phenyl]-2-tetradecylsulfanylacetamide	P	2.98	0.04	2.20	up
	12-Epinapelline	P	2.52	0.02	2.40	up
	Desacetyllevonantradol	P	2.25	0.04	2.16	up
	Dilauryl 3,3′-thiodipropionate	P	2.16	0.00	2.72	up
	Arginylserine	P	−1.32	0.03	1.95	down
	Asterinic acid	P	−1.46	0.01	2.42	down
	3-Acetylcalcicolin A	P	−1.67	0.05	1.80	down
	Bakkenolide H	P	−2.18	0.01	2.67	down
	Acebutolol	P	−3.43	0.04	2.46	down
HCB. vs. CK	CREATININE	P	3.21	0.00	2.94	up
	N-[2,6-Di(propan-2-yl)phenyl]-2-tetradecylsulfanylacetamide	P	2.47	0.01	1.96	up
	Dilauryl 3,3′-thiodipropionate	P	2.43	0.00	2.97	up
	n-Pentadecylamine	P	1.96	0.03	1.88	up
	hyocholic acid	P	1.93	0.00	1.89	up
	Tecomine	P	−3.00	0.02	1.73	down
	Lys Val Leu	P	−3.07	0.02	1.77	down
	Val Ile Leu	P	−3.18	0.04	1.58	down
	Ethyl N2-acetyl-L-argininate	P	−3.23	0.02	1.81	down
	Adenosine	P	−3.66	0.03	2.54	down
LCB. vs. CK	p-Coumaric acid sulfate	N	4.66	0.02	2.57	up
	3-(3-Hydroxyphenyl)propionic acid sulfate	N	4.28	0.02	2.84	up
	Dihydroferulic acid 4-O-sulfate	N	4.12	0.01	2.67	up
	Tichocarpol A	N	3.93	0.01	2.75	up
	Tricarballylic acid	N	2.11	0.02	3.12	up
	Ethyl gallate	N	−0.67	0.00	1.88	down
	Rhodomollein XIV	N	−0.78	0.03	2.31	down
	3,3-Dimethylglutaric acid	N	−1.05	0.03	2.22	down
	Isariotin E	N	−1.36	0.04	2.16	down
	3-anhydro-6-hydroxy-ophiobolin A	N	−1.53	0.02	2.76	down
MCB. vs. CK	Physagulin F	N	2.61	0.03	2.43	up
	4-Methoxyphenol sulfate	N	1.85	0.01	2.08	up
	Vielanin A	N	1.77	0.04	2.54	up
	3-(3-Hydroxyphenyl)propionic acid sulfate	N	1.70	0.04	1.26	up
	(7S)-Hydroprene	N	1.42	0.00	2.72	up
	2′-deoxyadenosine-5′-monophosphate	N	−2.30	0.02	2.39	down
	(4S)-4-Hydroxy-5-phosphonooxypentane-2,3-dione	N	−2.32	0.01	2.34	down
	Uridine 5′-monophosphate	N	−2.39	0.02	2.42	down
	GMP	N	−2.52	0.03	2.24	down
	Cytidine-3′-monophosphate	N	−2.53	0.03	1.84	down
HCB. vs. CK	p-Coumaric acid sulfate	N	4.55	0.00	2.42	up
	Dihydroferulic acid 4-O-sulfate	N	4.50	0.00	2.62	up
	Isoferulic acid 3-sulfate	N	4.42	0.02	2.16	up
	Tichocarpol A	N	3.96	0.00	2.53	up
	3-(3-Hydroxyphenyl)propionic acid sulfate	N	3.54	0.00	2.47	up
	Isariotin E	N	−1.58	0.01	1.63	down
	2′,3′-Dideoxycytidine-5′-monophosphate	N	−2.07	0.01	1.58	down
	Octaprenyl diphosphate	N	−2.25	0.04	1.21	down
	PA(14:0/22:6(4Z,7Z,10Z,12E,16Z,19Z)-OH(14))	N	−2.38	0.04	1.35	down
	Guanosine	N	−3.23	0.02	1.69	down

## Data Availability

The raw sequencing data, chromatographic data, and other experimental animal indicator test data generated in this study are part of ongoing follow-up work and mechanistic studies. Therefore, these data are not publicly available at this time. Researchers interested in accessing the data for non-commercial academic purposes may contact the corresponding authors by email.
